# Use of the ‘patient journey’ model in the internet-based pre-fitting counseling of a person with hearing disability: study protocol for a randomized controlled trial

**DOI:** 10.1186/1745-6215-14-25

**Published:** 2013-01-24

**Authors:** Vinaya KC Manchaiah, Dafydd Stephens, Gerhard Andersson, Jerker Rönnberg, Thomas Lunner

**Affiliations:** 1 Centre for Long Term and Chronic Conditions, College of Human and Health Sciences, Swansea University, Room 167, Glyndwr Building, Swansea, SA2 8PP, United Kingdom; 2 Linnaeus Centre HEAD, The Swedish Institute for Disability Research, Department of Behavioral Science and Learning, Linköping University, SE-58183, Linköping, Sweden; 3 Department of Psychological Medicine & Neurology, School of Medicine, Cardiff University, Cardiff, CF14 4XN, United Kingdom; 4 Department of Clinical Neuroscience, Division of Psychiatry, Karolinska Institutet, Stockholm, Sweden; 5 Eriksholm Research Centre, Oticon A/S, 20 Rørtangvej, DK-3070, Snekkersten, Denmark

**Keywords:** Hearing loss, Hearing impairment, Hearing disability, Patient journey, Counseling, Audiological rehabilitation, Internet

## Abstract

**Background:**

Hearing impairment is one of the most frequent chronic conditions. Persons with a hearing impairment (PHI) have various experiences during their ‘journey’ through hearing loss. In our previous studies we have developed a ‘patient journey’ model of PHI and their communication partners (CPs). We suggest this model could be useful in internet-based pre-fitting counseling of a person with hearing disability (PHD).

**Methods/Design:**

A randomized controlled trial (RCT) with waiting list control (WLC) design will be used in this study. One hundred and fifty eight participants with self-reported hearing disability (that is, score >20 in the Hearing Handicap Questionnaire (HHQ)) will be recruited to participate in this study. They will be assigned to one of two groups (79 participants in each group): (1) Information and counseling provision using the ‘patient journey’ model; and (2) WLC. They will participate in a 30 day (4 weeks) internet-based counseling program based on the ‘patient journey’ model. Various outcome measures which focuses on hearing disability, depression and anxiety, readiness to change and acceptance of hearing disability will be administered pre (one week before) and post (one week and six months after) intervention to evaluate the effectiveness of counseling.

**Discussion:**

Internet-based counseling is being introduced as a viable option for audiological rehabilitation. We predict that the ‘patient journey’ model will have several advantages during counseling of a PHD. Such a program, if proven effective, could yield cost and time-efficient ways of managing hearing disability.

**Trial registration:**

ClinicalTrials.gov Protocol Registration System NCT01611129.

## Background

The term ‘patient journey’ refers to the experiences patients go through during their disease and treatment. It is believed that understanding the ‘patient journey’ can help the clinician to gain an insight into the unique experiences of patients. In the last decade, with increased focus on patient-centered treatment approaches in healthcare, studies of the ‘patient journey’ have become popular. The ‘patient journey’ has been studied in various conditions including: Parkinson’s disease [1], locked-in syndrome [2], pertussis [3], gastrointestinal stromal tumors [4], and rheumatoid arthritis [5,6]. In our previous studies we developed ‘patient journey’ models for adults with gradual-onset [7,8] and sudden-onset [9] acquired hearing impairment.

**Figure 1:**
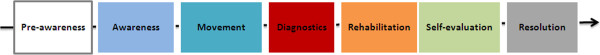
**‘Patient journey’ model of adults with gradual-onset acquired hearing impairment**[7]**.**

Figure 1 shows the ‘patient journey’ model of adults with gradual-onset acquired hearing impairment [7]. This model shows that there are seven main phases in this process, which include: (1) pre-awareness; (2) awareness; (3) movement; (4) diagnostics; (5) rehabilitation; (6) self-evaluation; and (7) resolution. Manchaiah *et al*. argued that this model could help the clinicians during history taking to understand at what stage the patient might be and then to tailor the way they speak to them [7].

### Pre-fitting counseling

There is a range of interventions focusing on the psychosocial needs of people with acquired hearing loss. For example, counseling-based aural rehabilitation [10]; active communication education (ACE) [11], rehabilitative online education [12] and cognitive behavioral self-help program [13]. However, pre-fitting counseling is mainly used for assessing and modifying the patient’s belief, motivation and expectations towards communication and to provide information about hearing loss and choice of interventions. Such counseling sessions could be very important in the audiological enablement process [14]. Even though pre-fitting counseling sessions could be potentially beneficial in various domains, there appears to be very little or no benefit in terms of the outcome of hearing aid fitting [15,16]. However, the main reason for pre-fitting counseling is to support the person with hearing impairment (PHI) in terms of their emotional and social needs, to assess and modify attitudes and motivations and to provide information about the choice of interventions [17], rather than focusing on hearing aid outcomes.

PHI go through various unforeseen consequences during their ‘journey’ through hearing loss. In a recent study by Laplante-Lévesque *et al*. it was identified that PHI mainly use their life experiences to describe their hearing help-seeking and hearing rehabilitation process rather than the interactions with clinicians [18]. It is believed that patients’ knowledge of the journey (that is, phases and stages they may go through) may reduce their anxiety and fear [19]. This is because the ‘patient journey’ model may relate to their lived experiences rather than focusing on the audiogram, causes of hearing loss, and hearing aids. In addition, the ‘patient journey’ model also presents them with the stages or the experiences they may go through in the future.

The ‘patient journey’ model considered above corresponds to the stages of change in the ‘transtheoretical model of change’ [20,21]. There have been various attempts in the literature to facilitate the stages of change in various domains including cessation of smoking, injury prevention, and weight loss. Manchaiah suggested that the use of health behaviour change models could be useful in facilitating help-seeking in patients with hearing loss [22]. For example, according to the health belief model (HBM) five main factors: (1) perceived severity; (2) perceived susceptibility; (3) perceived benefits; (4) perceived barriers; and (5) health motivation, may influence an individual’s decisions in proactive health behaviour [23]. We suggest that making the PHI aware of the ‘patient journey’ and in particular which stages they are currently in and what to expect later on, would increase their acceptance of hearing loss and readiness to change, hence positively facilitating stages of change in reaction to the hearing loss.

Moreover, considering the above, it is reasonable to expect that the ‘patient journey’ model can be used as a counseling tool to create awareness and educate PHI and their CPs during initial consultations. This may result in various advantages such as decreased emotional and social problems, decreased anxiety and depression, increased acceptance of hearing disability, and may positively facilitate stages of change through hearing disability, which are important factors in determining the success of audiological enablement/rehabilitation.

### Internet-based audiological rehabilitation

In recent years there has been an increase in the use of the internet for seeking health-related information. Cummings *et al*. suggested that online support groups in the form of a subscription distribution list can help people with hearing loss [24]. Laplante-Lévesque *et al*. used daily internet-based communications (in the form of Emails) in addition to the usual audiological service provided to people with hearing loss [25]. Their study demonstrated that an internet-based counseling program for new hearing aid users could be interactive and effective. Moreover, they found that each participant exhibited different behaviors and shared different thoughts during internet-based audiological counseling. More recently, Thorén *et al*., in a randomized controlled study evaluated the use of a rehabilitative online education program with discussion groups for hearing aid users [12]. They observed significant differences between pre and post counseling sessions in both groups, and the effects were maintained at six months follow-up. Overall, these studies suggest that the internet may be effectively used in audiological rehabilitation.

Considering the increased use of the internet by the general population, which increases flexibility for patients to participate in such programs in their own time and preferred place, and in the interest of reducing clinicians’ contact time, we will employ internet-based counseling in this study.

This paper describes the design of a randomized controlled trial to evaluate the effectiveness of the ‘patient journey’ model in the internet-based pre-fitting counseling of persons with hearing disability.

### Aim

The study is aimed at testing the hypotheses that, during the initial consultation of the PHD:

(1) Use of the ‘patient journey’ model as an internet-based counseling tool will result in decreased hearing-related emotional and social problems.

(2) Use of the ‘patient journey’ model as an internet-based counseling tool will result in decreased depression and anxiety.

(3) Use of the ‘patient journey’ model as an internet-based counseling tool will result in increased readiness to change (or positively facilitate stages of change through hearing disability).

(4) Use of the ‘patient journey’ model as an internet-based counseling tool will result in increased acceptance of hearing disability.

## Method

### Participant recruitment and study design

**Figure 2:**
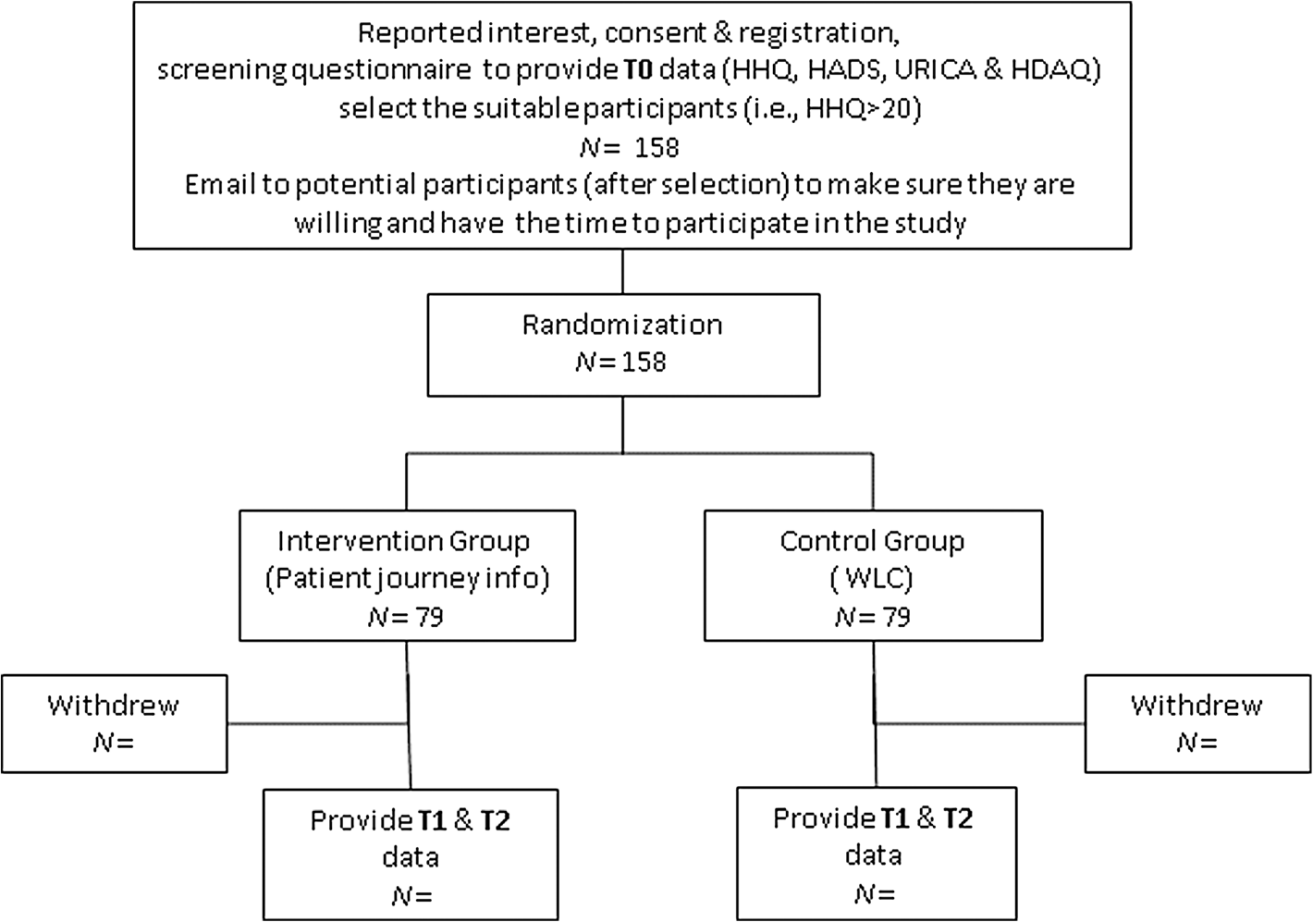
Flow chart showing the study design.

Ethical approval has been received from the Research Ethics Committee, College of Human and Health Sciences, Swansea University. A study advertisement will be made (in the United Kingdom) through various sources including local/national newspapers, hearing loss charity websites and mailing lists, inviting those with acquired hearing disability who have not yet had their hearing aids and who have internet access, to participate in this study. They will be given an information sheet describing the study and directing them to the website. In the first stage, interested participants will complete an informed consent form and the online questionnaires and the first 158 participants showing scores of over 20 in the Hearing Handicap Questionnaire (HHQ) will be recruited to participate in the study (that is, showing at least mild hearing disability). The HHQ scores can range from 12 to 60, from no hearing disability to a severe degree of hearing disability. In this study a wide range of people with hearing disability (that is, mild to severe degree) will be selected. However, a minimum score of over 20 was deemed appropriate to avoid the floor effect. The participants recruited will be randomized (randomization will be done independently of the researcher, by another person) to one of the two groups: (1) Information and counseling provision using the ‘patient journey’ model; and (2) Waiting list control (WLC). In addition, in the beginning of the study an Email with clear information about the study will be sent to willing participants to ensure that they have the time to participate in the study. Data will be collected using the various outcome measures discussed below pre and post counseling sessions. Figure 2 outlines the study design.

Inclusion criteria:

▪ Age over 18 years

▪ Noticing symptoms of hearing disability

▪ Access to internet

Exclusion criteria:

▪ Already using hearing aids

▪ HHQ score 20 or below

▪ Those with additional disabilities (for example, visual impairment, learning disability, dementia, and so on) which may affect individuals’ ability to participate in an internet-based program

### Intervention

Group one will be given ‘patient journey’ counseling which is based on previous studies [7,8,26], through an internet-based counseling protocol system. The focus of this would be on lived experiences of PHD rather than technical information such as the audiogram and hearing aids. This would involve four stages of designated internet sessions and additional tasks which the patients can complete in their own time. This program should be completed within 30 days. These sessions include: Stage 1 - introduction to the concept of the ‘patient journey’ and presenting to the participants a series of questions which may help them to explore their ‘journey’ through hearing loss; Stage 2 - the ‘patient journey’ model of PHI and two case examples will be presented. The PHD will be advised to compare their ‘journey’ to the model presented and identify the similarities and differences; Stage 3 - the ‘communication partners’ journey’ model will be presented and the PHD will be asked to consider how interactions between him/her and the CP may affect various things in the physical, mental and social domains; and Stage 4 - participants will be encouraged to think about how the PHI and CP may influence each other during their ‘journey’ through hearing loss, how they can overcome some of the difficulties they may be experiencing and to think about the potential benefits and the challenges from the audiological management.

Each stage will contain a short video (that is, one to two minutes) which briefly explains the tasks. However, the information presented in the video is also available in text to make sure equal access is provided to those who may find it difficult to understand speech in the video. Having information in different formats (for example, text and video) would help in ensuring that the information is presented in an interactive manner and that it engages the users in the activity. Throughout this process, the participants will be advised to reflect (a simple guide for reflection will be provided) and maintain notes. This reflection exercise is to explore the activity limitations and participation restrictions they have and also to explore the ways in which they can overcome them. In addition, there will be an Email contact with the researcher (who is also a trained audiologist) with whom they can communicate, update their progress with the intervention and seek further assistance.

The second group, which serves as the WLC, will complete the questionnaire at the beginning of the study and after 30 days will be provided with the same intervention as the other group. During the 30 days waiting period, the WLC will be advised to read generally about hearing loss and its treatment.

The study protocol has been registered in the http://ClinicalTrials.gov Protocol Registration System and the registration number is NCT01611129 (Study ID Number FAS-IT-03).

### Outcome measures

The main outcome measure used will be the Hearing Handicap Questionnaire (HHQ) and the secondary outcome measures include the Hospital Anxiety and Depression Scale (HADS), the University of Rhode Island Change Assessment Scale (URICA) and the Hearing Disability Acceptance Questionnaire (HDAQ).

HHQ is an instrument which has 12 questions scored on a 5-point Likert scale (never, to almost always) and provides a measure of personal and social effects - emotional distress and discomfort, social withdrawal, and general restriction of participation. HHQ was developed on the basis of a questionnaire by Hétu *et al*. [27] and an unpublished general health scale - Glasgow Health Status Inventory [28]. HHQ has a good Cronbach’s alpha of 0.95 for the emotional and 0.93 for the social scale [29].

HADS is an instrument for screening for anxiety and depression [30]. The HADS consists of 14 items divided into two subscales (anxiety and depression). Each item is scored from 0 to 3 (0 = not at all, 3 = most of the time) with a total score of 0 to 21 per subscale. In general, HADS has good reliability and acceptable sensitivity and specificity [31].

URICA is a stages of change measure consisting of four sub-scales: pre-contemplation, contemplation, action and maintenance [32,33]. The original URICA scale consists of 32-items. However, in this study we use a modified version (‘the problem’ is replaced by ‘the hearing problem’) consisting of 24-item scale which has also been used in a number of studies [34,35]. This modified version focuses on pre-contemplation, contemplation, and action stages of change. Each item is rated on a 5-point Likert scale (1 = strong disagreement, 5 = strong agreement). Each sub-scale measures specific aspects. For example, the pre-contemplation scale is assumed to capture unwillingness to change a problem behaviour or ignorance regarding the problem; contemplation items assess whether the person is seriously considering change and/or considering the pros and cons of not changing; action items assess whether the individual is engaging in change; and maintenance items assess the degree that change is integrated into a person’s life. In addition, the subscales are combined arithmetically (contemplation + action + maintenance – pre-contemplation), which provides a secondary readiness to change score which can be used to assess readiness to change at entrance to treatment. The readiness score can range from −2.00 to +14.00, with higher scores representing greater motivation to change. A recent study by Laplante-Lévesque *et al*. which investigated the usage of the URICA scale in adults with acquired hearing impairments seeking help for the first time, showed good construct, concurrent and predictive validity of the scale [36].

HDAQ has been designed based on the Tinnitus Acceptance Questionnaire (TAQ) which was developed by Westin *et al*. [37] in Sweden. This is a measure of experiential avoidance/acceptance and consists of 12 items. Each of the 12 items is rated on a 7-point Likert scale (1 = never true, 7 = always true).

### Sample size

HHQ is the primary outcome measure used for the power calculation. The results from a previous study suggest that the mean scores of HHQ for those with mild hearing loss who are seeking help for the first time are expected to be about an average of 26, with a standard deviation of 8 [36]. HHQ has been previously used in a few studies as predictive and outcome measure and shown a shift of around 2.5 to 5 points [11,38]. However, we expect to reduce the mean scores to 22 from 26 after the intervention (that is, reduction in perceived hearing disability by 4 points in HHQ). To detect this effect the sample size was calculated using PASS (NCSS, LLC, Kaysville, UT, USA) power analysis software (two-sample test, two-sided significance level, alpha of 0.05 and beta of 0.8), which showed that 66 participants per group are needed. However, considering the additional 20% loss to follow-up, the required sample size is 158 people in total, with 79 participants assigned to the control group and 79 participants to the intervention group.

### Blinding

Due to the nature of the study, it is not possible to blind the participants and the clinician. However, the allocation of participants to study groups will be done randomly using computer-generated numbers and with the help of another person (that is, researcher-blinded). However, the participants will be given unique reference numbers to keep the participants’ personal details blinded while analyzing the results.

### Co-intervention and compliance

Considering that the participants do not have any direct contact with the researcher (that is, the trained clinician), co-intervention cannot be avoided. However, participants will be requested not to participate in any interventions which may influence the results during the beginning of the study and will be questioned about this at the end by being asked a similar question after outcome measures have been collected. These notes may help us while analyzing the results. In relation to compliance with the intervention protocol, participants will be encouraged to complete the task after each session throughout this process. A reminder Email will be sent on a regular basis advising the participants about the recommendations made regarding intervention. Moreover, attempts will be made to classify the Email interactions with participants to understand at what phase each participant might be, so that the suggestions and recommendations made will be tailored accordingly.

### Data collection and analysis

Data will be collected via online questionnaires administered through the secure internet-based counseling protocol system one week pre intervention, one week post intervention and six month follow-up. To reduce attrition rate at follow-up, reminders will be sent by Email. However, if the participants decide not to participate further in the study, the reasons for their withdrawal will be recorded. Analysis of covariance will be made for T1 and T2 data with initial T0 measures (that is, pre and post counseling results) as the continuous predictor. Any missing data at T1 and T2 will be imputed with an assumption that values are missing completely at random (MCAR) and using an appropriate imputation method [39].

## Discussion

It is well documented that only a small percentage of people with hearing loss seek help and uptake amplification devices [40]. Moreover, it has been reported that on average people take about ten years before they decide to seek help [41]. There are various factors which can influence hearing loss help-seeking and hearing-aid uptake; however, studies, mainly from western countries, suggest that perceived hearing disability is known to be one of the important factors [42,43]. A recent study suggests that self-reported hearing problems (that is, perceived hearing disability) are more frequent than the hearing impairment measured through audiometric testing [44]. Also, those with self-reported hearing problems have increased likelihood of having hearing impairment [45]. Moreover, self-perceived hearing disability seems to be a better predictor of reduced quality of life than measured hearing impairment [46]. For this reason, audiological enablement/rehabilitation should be based on perceived difficulties rather than the severity or level of hearing impairment [47].

Studies indicate that participating in counseling-based aural rehabilitation would bring additional benefit in reducing perceived hearing disability than hearing aids alone [10]. A recent study conducted in Australia looked into actions taken by adults who failed telephone-based hearing screening [48]. The results indicated that those who had considered rehabilitation options (for example, hearing aids) are significantly more likely to seek professional help for their hearing impairment. These findings strongly add to our belief that knowing the ‘journey’ through hearing loss may better prepare the PHD to seek help. Moreover, the concept of ‘readiness management’ seems to have gained clinical interest when dealing with those who are seeking help or using hearing aids for the first time [49]. This process may involve information provision and modification of attitudes and expectations. Whilst such a program may not guarantee the successful rehabilitation of all cases, it may increase the likelihood of success.

Internet-based rehabilitation programs have recently started coming in to use and studies have shown promising results [11,25]. This internet-based counseling using the ‘patient journey’ model, is expected to benefit the people with perceived hearing difficulties in a number of ways including: reduced perceived hearing disability, decreased anxiety and depression, positive change in the stages of change and increased acceptance of hearing disability. This approach employs narrative-based medicine strategies to positively facilitate people with hearing difficulties. Each PHI may go through very unique experiences of hearing loss [8], however, the ‘patient journey’ model may provide a framework for the typical ‘journey’ of PHI [7], which may help them to focus on key stages while they think about their ‘journey’ through their hearing difficulties. It is important to note that the ‘journey’ may not be as linear in all the cases as is suggested in the typical model; however, we do expect that the participants will go through all of the main phases suggested in the model. Moreover, a recent qualitative study on shared decision-making in rehabilitative audiology emphasizes that PHI ‘wanted rehabilitative audiologists to hear their experiences and preferences and to tailor their interventions accordingly’ [50], which seems to support our approach in this study.

Whilst we expect to reduce perceived hearing disability through this intervention, there are very small chances that the opposite could happen in some cases. This is because thinking about the future challenges related to hearing problems may be overwhelming for some participants. For this reason, care must be taken during this process to boost positive aspects and to reduce negative aspects associated with their hearing problems. However, if proven effective, such a program could be a cost-effective and time-efficient program to help hundreds and thousands of people with perceived hearing difficulties.

Whilst efforts will be made to provide internet-based information in an interactive manner, no particular accommodation has been made for people with other disabilities such as visual impairment, learning disability, dementia, and so on, who may have difficulty understanding the information presented online. Moreover, one of the pre-requisites for the participants is having internet access. These criteria may result in the exclusion of some groups of people from this study. Moreover, one of the main predicted problems is in recruiting study participants, which will be managed by using appropriate advertisement methods.

## Trial status

Participant recruitment is complete and the trial is running during November to December 2012.

## Abbreviations

ACE: Active communication education; ANOVA: Analysis of variance; CP: Communication partner; HBM: Health belief model; HHQ: Hearing Handicap Questionnaire; HADS: Hospital Anxiety and Depression Scale: HDAQ, Hearing Disability Acceptance Questionnaire; MAR: Missing at random; PHD: Person with hearing disability; PHI: Person with hearing impairment; RCT: Randomized controlled trial; SRMI: Sequential regression multiple imputation; TAQ: Tinnitus Acceptance Questionnaire; URICA: University of Rhode Island change assessment scale; WLC: Waiting list control.

## Competing interests

The authors declare that they have no competing interests.

## Authors’ contributions

All the authors took part in the design of the study. VM is responsible for the overall operational aspects of the study including data collection, analysis and writing-up. TL, DS, JR and GA provided input to study design, development of internet-based counseling protocol and supervision of the study. All authors participated in the preparation of, and approved for publication, the final manuscript.
